# Heat stress effects on the genetics of growth traits in Thai native chickens (Pradu Hang dum)

**DOI:** 10.5713/ab.23.0165

**Published:** 2023-08-24

**Authors:** Wuttigrai Boonkum, Vibuntita Chankitisakul, Srinuan Kananit, Wootichai Kenchaiwong

**Affiliations:** 1 Department of Animal Science, Faculty of Agriculture, Khon Kaen University, Khon Kaen 40002, Thailand; 2 Network Center for Animal Breeding and Omics Research, Faculty of Agriculture, Khon Kaen University, Khon Kaen 40002, Thailand; 3 Small Ruminant Research Unit, Faculty of Veterinary Science, Mahasarakham University, Mahasarakham 44000, Thailand

**Keywords:** Absolute Growth Rate, Average Daily Gain, Body Weight, Heat Tolerance, Indigenous Chicken

## Abstract

**Objective:**

The objective of this study was to investigate the effect of heat stress on the growth traits and genetic parameters of Thai native chickens.

**Methods:**

A total of 16,487 records for growth traits of Thai native chickens between 2017 and 2022 were used in this study. Data included the body weight at birth, body weight at 4, 8, and 12 weeks of age (BW0, BW4, BW8, BW12), average daily gain during 0 to 4, 4 to 8, and 8 to 12 weeks of age (ADG0–4, ADG4–8, ADG8–12), absolute growth rate at birth, at 4, 8, and 12 weeks of age (AGR0, AGR4, AGR8, AGR12). The repeatability test day model used the reaction-norm procedure to analyze the threshold point of heat stress, rate of decline of growth traits, and genetic parameters.

**Results:**

At temperature and humidity index (THI) of 76, Thai native chickens began to lose their growth traits, which was the onset of heat stress in this study. The estimated heritability, genetic correlation between animal and heat stress effect, and correlations between the intercept and slope of the permanent environmental effects were 0.27, −0.85, and −0.83 for BW, 0.17, −0.81, and −0.95 for ADG, 0.25, −0.61, and −0.83 for AGR, respectively. Male chickens are more affected by heat stress than female chickens with a greater reduction of BW, ADG, and AGR, values equal to −9.30, −0.23, −15.21 (in males) and −6.04, −0.21, −10.10 (in females) gram per 1 level increase of THI from the THI of 76.

**Conclusion:**

The influence of thermal stress had a strong effect on the decline in growth traits and genetic parameters in Thai native chickens. This study indicated that genetic models used in conjunction with THI data are an effective method for the analysis and assessment of the effects of heat stress on the growth traits and genetics of native chickens.

## INTRODUCTION

Growth traits are one of the most economically important traits and are easily susceptible to environmental changes in poultry production systems [1–3]. Although several studies (nutrition and farm management) are ongoing to improve these traits under heat stress conditions [4–7], it has been found that heat stress problems cannot be solved sustainably. For this reason, it is essential to consider alternative methods that are more effective and more sustainable. The genetic approach is an alternative method and study of it is ongoing. Genetic methods are divided into two major types: molecular and quantitative genetic methods. In molecular genetics, in terms of marker-assisted selection, quantitative trait loci have been studied extensively in poultry, for example, in the naked neck, frizzle, and dwarf heat shock protein genes [8–11]. This approach has been applied successfully in commercial broilers and layers. Meanwhile, there are few studies of these genes in native chickens [12–14]. However, this approach may not be suitable in some areas due to the following limitations: it is not practical in large populations because it is expensive and laborious and requires high quality and large quantities of DNA [15]. Hence, another interesting method called the quantitative genetic approach should be more advantageous than the molecular method because of its ease of use in large populations, time from analysis to quick interpretation of results, and lower costs [16]. In addition, the quantitative genetics approach is concerned with the overall effects of all relevant genes regulating a trait [17], while the molecular genetics approach is concerned with individual genes or major genes or their specific functional genes only [15].

In the past, genetic improvement by the quantitative genetic approach of growth traits in chickens has not considered the effects of heat stress in the analysis process, resulting in high mortality in fast-growing chickens and extension of the fattening period [18]. In commercial chickens, the development of heat-tolerant chicken breeds may not be necessary because it is not a direct economic trait, and when there is a problem from hot and humid weather, farmers choose to fix it by modifying farm management as the priority. However, native chickens are different from commercial chickens, as most native chickens in all regions of the world are raised in open house systems that cannot control environmental temperature factors. At the same time, native chicken farmers do not have the budget to build chicken houses capable of controlling temperature and humidity or to install other equipment to help the chickens cool down. From all the above, relevant government agencies, including academics, should accelerate the development of chicken genetics to have good growth potential and heat tolerance.

Native chickens are raised in all regions of the world, play a vital role in the rural economies of many countries, and are considered a valuable genetic resource for use in the development of high-yielding breeds [19–21]. In some countries, indigenous chicken production is a matter of food security [22,23]. However, persistent temperature rise is currently a major threat to poultry production systems, especially in hot and humid climate areas. Sustainability in the near future may also affect the amount of food produced to meet the growing world population as well. Baumgard and Rhoads [24] mentioned that the optimal growth performance of chickens was prevented by heat stress, which is one of the crucial problems in animal production in many tropical countries. Beede and Collier [25] suggested reducing heat stress via genetic development for heat tolerance, which is a sustainable strategy. Genetic models combined with the temperature and humidity index (THI) are one of the tools from quantitative genetics that can be used to improve the genetic improvement of both growth and heat tolerance in chickens. From current information, if the ambient temperature is higher than the chicken’s thermal neutral temperature of 18°C to 24°C [26], the ability to dissipate heat from the body is lower, a sign that the chicken is undergoing heat stress. In general, under heat stress, chickens try to maintain a normal body temperature balance in various ways [27]. However, if the chicken is unable to get excess heat out of the body in time, chickens undergo heat stress manifesting as behavioral and physiological changes [28–30]. The effect of heat stress on growth is associated with decreased feed intake [31,32]. In broilers, THI above approximately 21°C resulted in an increase in body temperature to 1.7°C above the nominal body temperature for broilers (41°C), which significantly reduced the growth efficiency of broilers [33]. However, in tropical indigenous chickens, there have been very few reports of THI critical points on productivity. There is also a lack of important information such as genetic parameters for when chickens are under heat stress to plan for future losses.

Genetic parameters, such as heritability and genetic correlations, help quantify the degree to which genetics influences traits. High heritability suggests that a significant portion of the phenotypic variation is due to genetic factors, indicating that selection for those traits is likely adequate. By identifying traits with high heritability, breeders can focus on improving those traits through selective breeding. In addition, genetic parameters allow breeders to estimate the breeding values of animals. Breeding values are predictions of an animal’s genetic merit for specific traits based on its performance and the performance of its relatives. By selecting animals with high breeding values as parents, breeders can enhance the genetic potential of future generations, leading to improved traits in the population. Meanwhile, genetic parameters also provide an estimation of the response to selection. Response to selection measures how much genetic progress can be achieved in a specific trait within a given time frame. By understanding the genetic parameters, breeders can estimate the expected response to selection and make decisions on the intensity and direction of selection to maximize genetic gains. Finally, genetic parameters enable breeders to optimize breeding programs and design appropriate mating strategies. By considering the genetic parameters, breeders can determine the best combinations of animals for mating to achieve their breeding goals. For example, genetic correlations can help identify genetically linked traits, allowing breeders to improve multiple traits or simultaneously avoid unintended consequences of selection. For these reasons, this study aimed to investigate the effect of heat stress on the growth rate and genetic parameters of Thai native chickens.

## MATERIALS AND METHODS

This research was reviewed and approved by the Institutional Animal Care and Use Committee of Khon Kaen University based on the Ethics of Animal Experimentation of the National Research Council of Thailand (No. IACUC-KKU-135/64). The study was carried out on the experimental farm of the Network Center for Animal Breeding and Omics Research, Faculty of Agriculture, Khon Kaen University, Thailand.

### Animals and management

A total of 16,487 records for body weight (BW) of 4,120 Thai native chickens (Pradu Hang dum) (2,100 males and 2,020 females) from six generations between 2017 and 2022 were used in this study. Pedigree data were constructed by tracing back all generations of ancestors and included 17,035 individuals born between 2016 and 2021. Data included the BW at birth, body weight at 4, 8, and 12 weeks of age (BW0, BW4, BW8, BW12), average daily gain (ADG) during 0–4, 4–8, and 8–12 weeks of age (ADG0–4, ADG4–8, ADG8–12), absolute growth rate (AGR) at birth, at 4, 8, and 12 weeks of age (AGR0, AGR4, AGR8, AGR12) were collected and are shown in Table 1. After hatching, the chickens were weighed individually and an identification number was attached on the leg until four weeks of age, followed by wing banding to keep their BW records. All chickens were vaccinated for infectious bronchitis, Newcastle disease, and fowl pox according to the chicken vaccination program. Chickens were provided water and fed *ad libitum* using standard commercial broiler feed. Feed was divided into two formulas according to the age of the chickens. From hatching to four weeks of age, the feed consisted of 21% crude protein (CP) and 3,000 kcal metabolizable energy (ME/kg), followed by a grower feed of 19% CP and 2,900 kcal ME/kg. This feed was used until the end of the experiment at 12 weeks of age. All chickens were raised in open-air housing. The photo schedule consisted of two stages: the first stage was from hatching to four weeks of age with 24 h light/0 h dark using warming with a 100-watt lamp; the second stage was from after 4 to 12 weeks of age with natural light.

### Climatic conditions

Climate data were obtained from the meteorological center closest to the chicken farm (3 km distance). The weather information included daily temperature and relative humidity (RH) recorded every 3 hours, which were used to calculate the THI based on the formula [34]:


THI=(1.8×Temp+32)-(0.55-0.0055×RH)(1.8×Temp-26)

where Temp is the average temperature in degrees Celsius and RH is the average relative humidity as a percentage. Mean THI for the four weeks before the weigh-in for individual birds of each age was used to determine the heat stress threshold and assess genetic parameters. For THI calculation, average THI values from day 1 to 21 of incubation, average THI values from day 1 to 28 before the weigh-in, average THI values from day 29 to 56 before the weigh-in, and average THI values from day 57 to 84 before the weigh-in, which were used with the BW at birth (BW0), BW at 4, 8, and 12 weeks of age (BW4, BW8, and BW12), respectively. The average THI of the ADG and AGR characteristics was computed in the same of BW.

### Statistical analysis and growth curve estimation

The BW and ADG data were recorded from the experimental farm while the AGR data were calculated using the Gompertz function [3,35]. The AGR was also used as another observation value in this study to estimate the genetic parameters for heat stress. The steps of individual AGR analysis are as follows. Step 1: The Gompertz model by Marquardt method was performed by the NLIN procedure of SAS 9.2 software to estimate the growth curve of chicken, which by estimating the initial parameter values (*α*, *β*, *γ*) where *α* was the asymptotic live BW (grams), *β* was the log-function for the proportion of the asymptotic mature weight to be gained after hatch (t = weeks), and *γ* was a constant scale which is proportional to the overall growth rate (weeks). This step gave us the average growth curve parameters of the population. Step 2: To analyze individual AGR, the initial parameter values from Step 1 were defined as prior numbers to estimate individual *α*, *β*, *γ* by taking the first derivative then the individual AGR was calculated based on this equation *αβγe^(^*^−^*^βe^*^−^*^γt)^e*^−^*^γt^.*

All data were verified before genetic analysis using the Proc UNIVARIATE procedure by SAS v.9.0 program to examine data distribution, including assessing normality, homogeneity of variance, and checking data outliers (±3 standard deviation was defined as outlier). Statistical differences were compared by sex using the post hoc test in the generalized linear model for an unbalanced analysis of variance (general linear model procedure) using the SAS package. The decrease in initial growth characteristics caused by the influence of temperature and RH was analyzed using a simple regression analysis method.

### Estimation of genetic parameters

The repeatability test-day model used the reaction-norm procedure proposed by Bohmanova et al [36] to estimate the threshold point of heat stress and genetic parameters. Different thresholds of the THI, from 72 to 80 (THI72 to THI80), were tested in the model. The THI function was created to estimate the decline in BW, ADG, and AGR traits under heat stress conditions. The repeatability test-day model, THI function, and variance-covariance structure matrix used in this study were defined as follows:

The repeatability test day model used was:


$$y_{ijkl}={CG}_{i}+{Age}_{j}+{SEX}_{k}\;[f(THI)]+a_{0l}+a_{1l}\;[f(THI)]+p_{0l}+p_{0l}\;[f(THI)]+e_{ijkl}$$

where *y_ijkl_* is the observation value of the BW, ADG, and AGR for chicken l in contemporary group between hatch and generation (*CG*) class i, age (*Age*) class j, sex (*SEX*) class k; *CG_i_* is the fixed effect of the contemporary group (hatch and generation) i; *Age_j_* is the fixed effect of chicken age j; *SEX_k_* is the fixed effect of chicken sex k, which is nested with the THI function ([*f*(*THI*)]) to describe the change of BW, ADG, and AGR traits in males and females (look at slope of the regression) according to the change in THI values; *a_0l_* is the random additive genetic effects without consideration of heat stress (called “intercept”); *a_1l_* is the random additive genetic effects for heat stress (called “slope”); *p_0l_* is the random permanent environmental effects without consideration of heat stress; *p_1l_* is the random permanent environmental effects of heat stress; *e_ijkl_* is the random residual effect; *f*(*THI*) is a function of the THI.

The temperature and humidity function used was:


$$f(THI)=\{\begin{array}{ll} 0, & THI\leq THI_{threshold}\;(no\;heat\;stress)\\ THI-THI_{threshold}, & THI>THI_{threshold}\;(heat\;stress) \end{array}$$

The THI included in the repeatability test day model were set at various critical values or threshold points. Different thresholds, at 72 (THI72), 74 (THI74), 76 (THI76), 78 (THI78), and 80 (THI80) of THI, were tested in the model. The best fitting degree of THI was considered from three statistical criteria: the lowest minus twice the logarithm of the likelihood (−2logL), Akaike’s information criterion (AIC), and Bayesian information criterion (BIC).

The variance-covariance structure used was:


$$Var\left[\begin{array}{c} a_{0}\\ a_{1}\\ p_{0}\\ p_{1}\\ e \end{array}\right]=\left[\begin{array}{ccccc} A\sigma_{a0}^{2} & A\sigma_{a01} & 0 & 0 & 0\\ A\sigma_{01} & A\sigma_{a1}^{2} & 0 & 0 & 0\\ 0 & 0 & I\sigma_{p0}^{2} & I\sigma_{p01} & 0\\ 0 & 0 & I\sigma_{p01} & I\sigma_{p1}^{2} & 0\\ 0 & 0 & 0 & 0 & I\sigma_{e}^{2} \end{array}\right]$$

where $\sigma_{a0}^{2}$ is additive genetic variance without consideration of heat stress (called “intercept”); $\sigma_{a1}^{2}$ is additive genetic variance for heat stress (called “slope”); $\sigma_{p0}^{2}$ is permanent environmental variance without consideration of heat stress; $\sigma_{p1}^{2}$ is permanent environmental variance of heat stress; $\sigma_{e}^{2}$ is residual variance; *A* is the numerator relationship matrix; and *I* is an identity matrix.

Variance components were estimated with the average information restricted maximum likelihood (AI-REML) algorithm using AIREMLF90 program [37]. The heritability (this value is a statistical measure that quantifies the extent to which genetic variation contributes to phenotypic variation within a population) and permanent environmental variance (this value refers to the portion of phenotypic variation in a trait consistently influenced by non-genetic factors and remains constant across an individual’s lifetime) of the BW, ADG, and AGR under hot-humid climates were estimated by Ravagnolo et al [38] with the following equation:


$$\begin{array}{c} h^{2}=\frac{\sigma_{a0}^{2}+\sigma_{a1}^{2}+2\sigma_{a01}}{\sigma_{a0}^{2}+\sigma_{a1}^{2}+2\sigma_{a01}+\sigma_{p0}^{2}+\sigma_{p1}^{2}+2\sigma_{p01}+\sigma_{e}^{2}}\\ pe^{2}=\frac{\sigma_{p0}^{2}+\sigma_{p1}^{2}+2\sigma_{p01}}{\sigma_{a0}^{2}+\sigma_{a1}^{2}+2\sigma_{a01}+\sigma_{p0}^{2}+\sigma_{p1}^{2}+2\sigma_{p01}+\sigma_{e}^{2}} \end{array}$$

Genetic correlations (*r_g_*) between the intercept (the intercept of the random additive effect indicates the animal’s ability to produce BW, ADG, and AGR in thermoneutral conditions) and slope (the slope of the additive genetic effect describes the animal’s sensitivity to heat stress; it represents the change in BW, ADG, and AGR per THI unit increase above a given threshold). These values refer to the genetic components of two or more traits. It measures the extent to which the same genes or sets of genes influence multiple traits simultaneously. The correlations between the intercept and slope of the permanent environmental effects (*r_p_*) were calculated as follows:


$$r_{g}=\frac{COV\sigma_{a0,a1}}{\sqrt{\sigma_{a0}^{2}*\sigma_{a1}^{2}}},r_{p}=\frac{COV\sigma_{p0,p1}}{\sqrt{\sigma_{p0}^{2}*\sigma_{p1}^{2}}}$$

## RESULTS

### Body weight description

The BW, ADG, and AGR of Thai native chicken (Pradu Hand dum) is presented in Table 1. Male chickens had higher all growth traits than female chickens at all ages, but there was a significant difference from four weeks of age onward (p<0.05). The percentage of BW of male chickens was higher than that of female chickens as follows: 8.20%, 18.75%, 25.31%, and 29.41% at BW0, BW4, BW8, and BW12, respectively. In addition, when considering the coefficient of variation percentages, these values showed that Thai native chickens, both males and females, had good BW consistency from 4 weeks of age onward, which indicated that feed intake and management were similar throughout the chicken house. At the same time, the values of ADG0–4, ADG4–8, and ADG8–12 in males (6.1, 16.6, 17.6 g/d) were significantly higher than those in females (5.7, 13.5, 14.1 g/d) (p<0.05), according to AGR4, AGR8, and AGR12 in males (84.07, 128.36, 111.93 g/wk) were also significantly higher than females (73.87, 97.64, 93.49 g/wk) (p<0.05).

### Climatic conditions

**Figure 1:**
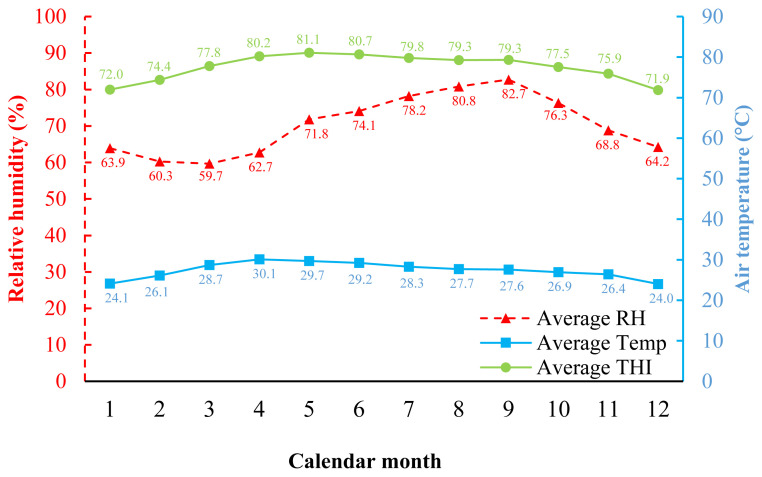
Average temperature and humidity index (THI), relative humidity (RH), and air temperature (Temp) during experimental period (2017 through 2022) in Khon Kaen province, Thailand.

In Figure 1, during 2017 to 2022, which is the experimental period, the climate of Khon Kaen was hot and humid throughout the year. The highest temperature was found from March to June (28.7°C to 30.1°C), which corresponds to the summer season, while the lowest temperature was found during November to February (24.0°C to 26.4°C), which corresponds to the winter season in Thailand. The percentage of RH was greater than 59% throughout the year, and when calculated in terms of average THI, it was in the range of 71.9 to 81.1.

### Effects of temperature and humidity index on growth traits

**Figure 2:**
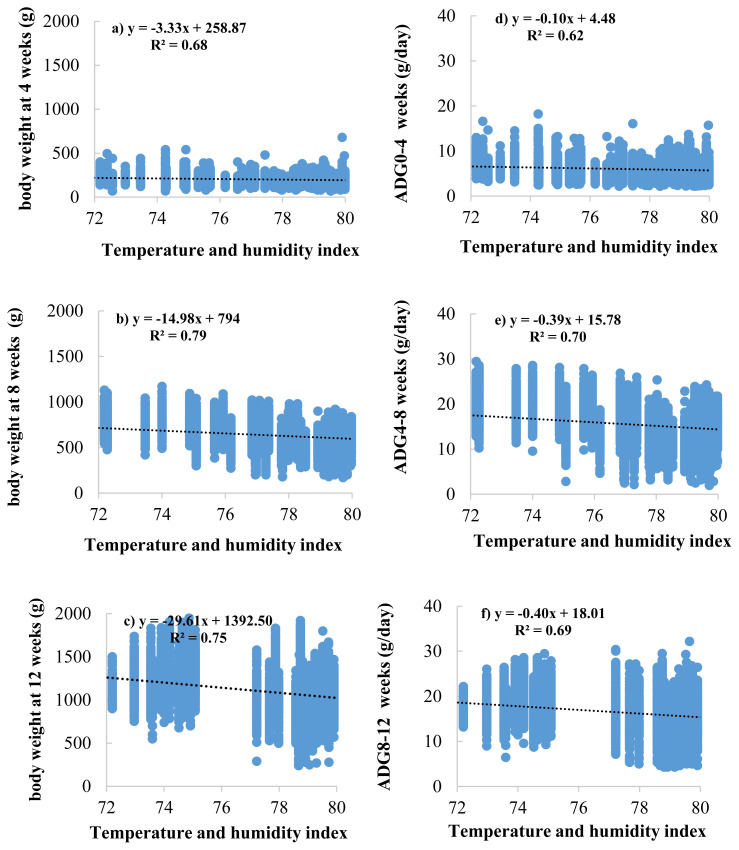
Regression analysis between temperature and humidity index (THI) and body weight at 4 (a), 8 (b), and 12 (c) weeks of age and average daily gain (ADG) during 0 to 4 (d), 4 to 8 (e), and 8 to 12 (f) weeks of age in Thai native chickens (Pradu Hang dum).

**Figure 3:**
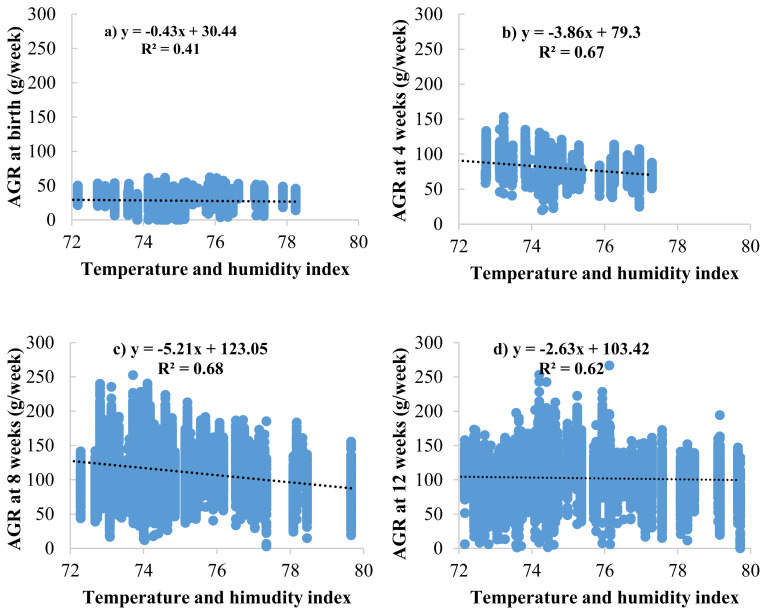
Regression analysis between temperature and humidity index (THI) and absolute growth rate (AGR) at birth (a), 4 (b), 8 (c), and 12 (d) weeks of age in Thai native chickens (Pradu Hang dum).

The effects of the THI on BW and ADG are presented in Figure 2. The results showed that THI increased, and BW decreased at 4, 8, and 12 weeks of age (BW4, BW8, and BW12) with values of −3.33, −14.98, and −29.61 g/THI, respectively (Figure 2a, 2b, and 2c). Meanwhile, the effects of THI on ADG resulted in the same direction as those in BW, ADG during 0 to 4, 4 to 8, and 8 to 12 weeks of age (ADG0–4, ADG4–8, and ADG8–12) were decreased with values of −0.10, −0.39, −0.40 gram/d/THI, respectively (Figure 2d, 2e, and 2f). At the same time, the effects of THI on AGR are presented in Figure 3. The results showed AGR at 8 weeks of age, and chickens were the most affected by heat stress as determined by the most significant decrease in AGR value (−5.21 gram/THI) (Figure 3c) compared to other ages, followed by 4 weeks (−3.68 gram/THI) (Figure 3b) and 12 weeks (−2.63 gram/THI) (Figure 3d) of age, respectively. The beginning of heat stress is assessed by the accuracy of the genetic assessment and the validity of the model when considering the lowest −2logL value is THI72 to THI80. However, from the area average THI data (Figure 1), it was found that throughout the year, the average THI was higher than THI76. However, from the area average THI data (Figure 1), it was found that throughout the year, the average THI was higher than THI76. In this study, the threshold point in Thai native chickens, the critical point of growth traits (BW, ADG, and AGR), was THI76.

### Estimated genetic parameters

The estimated variance components and genetic parameters of the BW, ADG, and AGR by the repeatability test day model with the THI function are presented in Table 2 and Table 3. In this study, the indicated threshold point of heat stress was found at a THI of 76 based on the lowest −2logL, AIC, and BIC. All variance component estimates increased with increasing THI values. The estimated heritability (h^2^) varied from 0.26 to 0.45 for BW, 0.26 to 0.34 for ADG, and 0.25 to 0.26 for AGR, while the permanent environmental variances (pe^2^) were higher than heritability and varied from 0.23 to 0.34 for BW, 0.02 to 0.08 for ADG, and 0.32 to 0.34 for AGR in the range of THI72 to THI80. The genetic correlations (r_g_) between the intercept and slope were −0.90 to −0.55 for BW, −0.87 to −0.57 for ADG, and −0.61 to −0.55 for AGR, and the correlations (r_p_) between the intercept and slope of the permanent environmental effects were −0.87 to −0.83 for BW, −0.96 to −0.67 for ADG, and −0.86 to −0.79 for AGR. The rate of BW, ADG, and AGR decline at a THI of 76 in male chickens (−9.30, −0.23, −15.21 gram per THI unit increase) was greater than that in female chickens (−6.04, −0.21, −10.10 gram per THI unit increase). One thing that can be seen from this result is that the higher the THI, the more the growth traits decrease as well.

## DISCUSSION

The BW and ADG of male Thai native chickens was higher than that of female Thai native chickens, consistent with several previous studies, such as [39–41]. However, when considering the average BW between Thai native chickens in this study and other Thai native chicken breeds, it was found that Thai native chickens in this study had a significantly higher BW, as in Lueng Hang Kao Kabinburi [42] in Chee [43]. The explanation for such results likely comes from continuous and effective genetic improvement planning for the growth of Thai native chickens. In fact, male chickens have a higher growth rate than female chickens, which means that males require a shorter rearing period than females to meet market demands. Accordingly, male chickens are also subjected to more heat stress than female chickens because they require more energy, and this can lead to an increased metabolic rate, which produces more internal heat. At the same time, if the temperature of the environment is also high, then heat stress occurs faster and increases the intensity. In addition, male chickens are often raised for meat production and are genetically selected for fast growth and high meat yield. This can lead to a higher metabolic rate and a decreased ability to cope with heat stress, making them more susceptible to heat stress. In addition, Thailand’s climate has high temperatures and humidity throughout the year, resulting in higher THI values. Therefore, compared to previous studies with lower temperature and humidity values than in this study, it is possible that Thai native chickens may also be subject to constant heat stress.

The results of AGR were consistent with those of BW and ADG. However, the important reasons for using AGR in this study are: i) AGR records provide a more detailed and dynamic representation of the growth patterns of poultry compared to BW records alone. Instead of relying solely on BW measurements at specific time points, growth rate records quantify the rate at which an individual gains weight over a particular period. This information allows for a better understanding of growth trajectories and patterns, including periods of rapid growth, growth plateaus, or potential growth abnormalities. ii) AGR records can help identify variations in growth performance at an early stage. Monitoring the weight gain rate over time makes detecting deviations or inconsistencies in growth patterns easier. This early detection enables prompt interventions or adjustments in management practices, such as adjusting feed rations, addressing health issues, or implementing targeted selection strategies to improve growth performance. iii) These records provide valuable data for estimating genetic parameters related to growth traits in poultry. By quantifying the rate of weight gain, it becomes possible to estimate the genetic components associated with growth rates more accurately. These estimates are essential for breeding programs focused on improving growth performance and can contribute to selecting superior individuals with higher growth potential. Finally, AGR records allow for better differentiation of the genetic potential for growth among individuals within a population. There needs to be more than BW records to provide a complete picture of the underlying growth abilities of poultry. By considering growth rates, it becomes possible to distinguish between individuals with similar BWs but different growth rates, thus enabling more precise selection decisions for genetic improvement.

The moderate to high heritability (0.26 to 0.45) for BW, and (0.25 to 0.26) for the AGR traits indicated that genetic selection by the traditional method through selection could be achieved, except in the ADG trait, which might require molecular genetic analysis due to its low heritability. The heritability values in this study were in agreement with those reported in previous studies; for example, the heritability of AGR in purebred and crossbred Shee Thai native chickens was 0.23 and 0.18 [3], respectively, varied from 0.10 to 0.25 in Korean native chickens [44], and was valued at 0.29 in Japanese quail [45]. In general, the heritability estimates for growth traits throughout life vary in native chickens and depend on the population [42,44]. Previous studies in poultry have shown decreased heritability estimates of weight gain with increasing age [46,47]. The heritability value in this study indicated that approximately 25% of the variation in the trait being studied can be attributed to genetic factors, while the remaining 75% of the variation is due to nongenetic factors, such as environmental factors and chance [17]. The heritability value can also be useful in animal breeding programs, where it is used to predict the potential genetic improvement that can be achieved through selective breeding. In addition, the heritability value can be used in genetics to estimate the likelihood that a particular trait or disease is inherited from a parent or other relative [48].

The permanent environmental variance was higher than the heritability in BW and AGR traits, reflecting that environmental factors are more important than genetic factors. The reduction in animal productivity is a consequence of changes in physiology and behavior in response to heat stress, which has been reported in several studies [49,50]. Therefore, intensive management is needed to promote better growth performance, including nutritional strategies such as restricted feeding, and maintaining feed intake, choice feeding from different feed ingredients rich in protein or in energy, electrolytic and water balance, and micronutrient supplementation [11,51]. Meanwhile, the genetic correlation estimates between regular conditions and heat stress were very high, suggesting that chickens with a high growth rate have low heat tolerance. Hence, selecting chickens for a high growth rate would lead to chickens with greater susceptibility to heat stress [33,52,53]. Although the genetic correlation is very large, the combined selection of growth rate and heat tolerance using the breeding value index is possible [38].

After the chickens reached heat stress at a THI of 76, the growth traits decreased. This finding suggests that heat stress negatively affects growth ability. The decrease in chicken growth rates because of heat stress is caused by decreased feed intake and increased drinking during heat stress [31–33]. Heat stress can increase chickens’ metabolic rate, which leads to an increase in their body temperature. When chickens eat, heat is generated in their bodies through the process of digestion. Eating less helps to reduce the heat load on the chickens’ bodies and can help to regulate their body temperature. At the same time, drinking more water helps chickens regulate their body temperature by increasing evaporative cooling through panting and sweating. When chickens pant, water is evaporated from their respiratory surfaces, which cools their body temperature. Drinking more water helps to increase the amount of water available for evaporation and cooling, which helps to reduce the heat stress on their bodies. Heat stress can also decrease the appetite of chickens by making them feel lethargic and less interested in eating. This decrease in appetite may be a response to the stress on their bodies, as the energy required for digestion and processing of feed can add to the metabolic load on their systems. In summary, heat stress can cause chickens to eat less food and drink more water to regulate their body temperature and cope with the stress on their bodies. It is important to provide chickens with access to cool water and shade during periods of heat stress to help them cope with these conditions.

The growth traits decrease from heat stress in terms of the average rate of decline of the BW, ADG, and AGR trait was greater effect in male than in female chickens at THI threshold (THI76) (−9.30, −0.23, and −15.21 and −6.04, −0.21, and −10.10 g/THI unit increase, respectively), similar to what has been reported previously [12,54]. Indeed, those studies indicate higher heat tolerance in Thai native chickens than in commercial broiler strains when reared in hot temperatures. Therefore, the use of genetics of native chickens may also be considered in the development of a new chicken genetic line. The additive variance and permanent environmental variance of the effect of heat stress increased sharply after the THI of 76. These data indicate that native chickens express genetic factors that allow them to cope with heat stress. There was decreased heritability of these factors at the higher THI, and thus, animal selection using genetics is not precise.

## CONCLUSION

Heat stress is a significant problem as it leads to reduced growth in poultry, particularly in tropical areas. Therefore, there is a need to develop native chicken genetic lines that can tolerate tropical climates without compromising growth performance in the slightest. The results of this study can be applied in farm management planning when chickens suffer from heat stress when the THI value is greater than 76. In addition, the problem of heat stress is likely to increase further. Therefore, chicken breeding programs should be considered to enhance genetic production potential and adapt to harsh climatic conditions. The new insight from this study is a unique genetic model that can be quickly and easily applied to a large dataset.

## Figures and Tables

**Table 1:** Statistical descriptive analysis of growth traits (body weight, average daily gain, and absolute growth rate) in Thai native chickens (Pradu Hang dum)

Traits^1)^	Male chicken^2)^					Female chicken^2)^				
	
	Mean	SD	Min	Max	CV	Mean	SD	Min	Max	CV
BW0	33.0^a^	3.5	20	46	10.61	30.5^a^	3.5	20	41	11.48
BW4	285.0^a^	7.9	150	420	2.77	240.0^b^	7.8	100	380	3.25
BW8	747.5^a^	48.8	600	895	6.53	596.5^b^	35.1	478	715	5.88
BW12	1,650.0^a^	67.0	1,450	1,850	4.06	1,275.0^b^	45.0	1,150	1,400	3.53
ADG0–4	6.1^a^	1.9	2.2	18.2	30.9	5.7^a^	1.7	2.4	15.0	29.7
ADG4–8	16.6^a^	4.3	5.0	29.5	25.9	13.5^b^	3.1	2.0	27.1	22.9
ADG8–12	17.6^a^	4.2	6.1	37.1	23.9	14.1^b^	3.2	4.1	27.5	22.7
AGR0	34.67^a^	12.51	0.95	59.73	38.23	32.76^a^	10.36	44.86	0.67	29.88
AGR4	84.07^a^	18.02	4.85	230.66	21.43	73.87^b^	13.37	153.00	3.48	18.10
AGR8	128.36^a^	37.01	7.73	246.09	28.83	97.64^b^	25.56	219.85	6.64	26.18
AGR12	111.93^a^	26.45	9.31	266.53	23.63	93.49^b^	23.68	253.02	7.72	25.33

1) BW0, BW4, BW8, and BW12 = body weight at birth, at 4, 8, and 12 weeks of age (g); ADG0–4, ADG4–8, and ADG8–12 = average daily gain during 0 to 4, 4 to 8, and 8 to 12 weeks of age (g/d); AGR0, AGR4, AGR8, and AGR12 = absolute growth rate at birth, at 4, 8, and 12 weeks of age (g/wk).

2) Mean, average value; SD, standard deviation; Min, minimum value; Max, maximum value; CV, coefficient of variation

a,b Means with different letters between chicken sex are different (p<0.05).

**Table 2:** Variance components, genetic parameters, model statistic criteria, and rate of decline of body weight and average daily gain in Thai native chickens (Pradu Hang dum) at various temperature and humidity indices

Items^1)^	Body weight					Average daily gain				
	
	THI72	THI74	THI76	THI78	THI80	THI72	THI74	THI76	THI78	THI80
Parameters										
V_a0_	11,110.00	9,137.70	6,831.50	3,771.70	3,467.00	1.53	1.25	1.02	0.69	0.52
V_a1_	146.43	195.71	283.15	304.82	800.99	0.03	0.04	0.06	0.09	0.11
Cov_a0,a1_	−1,159.20	−1,193.10	−1,188.40	−743.10	−1,050.10	−0.18	−0.18	−0.20	−0.18	−0.13
V_p0_	6,479.90	4,926.30	4,773.60	2,823.80	822.43	0.50	0.44	0.34	0.13	0.06
V_p1_	131.73	174.20	312.90	329.81	213.59	0.01	0.02	0.04	0.06	0.06
Cov_p0,p1_	−811.96	−780.96	−1,015.70	−584.05	382.05	−0.08	−0.10	−0.11	−0.08	−0.04
V_e_	6,680.00	6,493.00	6,229.70	6,772.10	6,972.10	4.59	4.52	4.44	4.48	4.55
h^2^ (±SE)	0.45±0.02	0.43±0.02	0.37±0.02	0.26±0.02	0.26±0.03	0.23±0.02	0.20±0.02	0.17±0.02	0.13±0.02	0.10±0.03
pe^2^ (±SE)	0.26±0.01	0.23±0.01	0.25±0.01	0.34±0.02	0.33±0.03	0.08±0.01	0.07±0.01	0.06±0.01	0.02±0.02	0.02±0.03
r_g_	−0.90	−0.89	−0.85	−0.58	−0.55	−0.87	−0.85	−0.81	−0.71	−0.57
r_p_	−0.87	−0.84	−0.83	−0.83	−0.86	−0.96	−0.95	−0.95	−0.88	−0.67
Rate of decline (gram/THI)										
Male	0	0	−9.30	−28.00	−36.77	0	0	−0.23	−0.47	−0.94
Female	0	0	−6.04	−18.12	−30.20	0	0	−0.21	−0.42	−0.83
Statistic criteria										
−2logL	919	473	0	616	968	528	275	0	251	515
AIC	919	473	0	616	968	528	275	0	251	515
BIC	919	473	0	616	968	528	275	0	251	515

THI, temperature and humidity indices; V_a0_, additive variance of animal; V_a1_, additive variance of heat stress effect; Cov_a0a1_, covariance between additive variance of animal and heat stress effect; V_p0_, permanent environmental variance of animal; V_p1_, permanent environmental variance of heat stress effect; Cov_p0p1_, covariance between permanent environmental variance of animal and heat stress effect; V_e_, residual variance; h^2^, heritability; SE, standard error; r_g_, genetic correlation between animal and heat stress effect; r_p_, correlations between the intercept and slope of the permanent environmental effects; −2logL, minus twice the logarithm of the likelihood; AIC, Akaike’s information criterion; BIC, Bayesian information criterion.

**Table 3:** Variance components, genetic parameters, model statistic criteria, and rate of decline of the absolute growth rate (AGR) in Thai native chickens (Pradu Hang dum) at various temperature and humidity indices (THI)

Items	Absolute growth rate				

	THI72	THI74	THI76	THI78	THI80
Parameters					
V_a0_	602.48	627.22	644.76	658.50	672.20
V_a1_	15.31	18.33	25.72	28.34	33.09
Cov_a0,a1_	−58.38	−64.12	−78.44	−79.15	−82.75
V_p0_	776.62	789.12	838.44	876.62	893.46
V_p1_	9.69	9.68	10.55	16.12	19.23
Cov_p0,p1_	−68.58	−72.44	−78.12	−98.71	−112.55
V_e_	846.63	846.63	846.63	846.63	846.63
h^2^ (±SE)	0.25±0.02	0.26±0.02	0.25±0.02	0.26±0.02	0.26±0.03
pe^2^ (±SE)	0.33±0.01	0.32±0.01	0.34±0.01	0.34±0.02	0.33±0.03
r_g_	−0.61	−0.60	−0.61	−0.58	−0.55
r_p_	−0.79	−0.83	−0.83	−0.83	−0.86
Rate of decline of AGR (gram/THI)					
Male	0	0	−15.21	−21.45	−34.41
Female	0	0	−10.10	−16.21	−28.95
Statistic criteria					
−2logL	4	21	0	59	81
AIC	4	21	0	59	81
BIC	4	21	0	59	81

THI, temperature and humidity indices; V_a0_, additive variance of animal; V_a1_, additive variance of heat stress effect; Cov_a0a1_, covariance between additive variance of animal and heat stress effect; V_p0_, permanent environmental variance of animal; V_p1_, permanent environmental variance of heat stress effect; Cov_p0p1_, covariance between permanent environmental variance of animal and heat stress effect; V_e_, residual variance; h^2^, heritability; SE, standard error; r_g_, genetic correlation between animal and heat stress effect; r_p_, correlations between the intercept and slope of the permanent environmental effects; AGR, absolute growth rate; −2logL, minus twice the logarithm of the likelihood; AIC, Akaike’s information criterion; BIC, Bayesian information criterion.

## References

[1] Wasti S, Sah N, Mishra B (2020). Impact of heat stress on poultry health and performances, and potential mitigation strategies. Animals.

[2] Perini F, Cendron F, Rovelli G, Castellini C, Cassandro M, Lasagna E (2021). Emerging genetic tools to investigate molecular pathways related to heat stress in chickens: a review. Animals.

[3] Boonkum W, Duangjinda M, Kananit S, Chankitisakul V, Kenchaiwong W (2021). Genetic effect and growth curve parameter estimation under heat stress in slow-growing Thai native chickens. Vet Sci.

[4] Madkour M, Salman FM, El-Wardany I (2022). Mitigating the detrimental effects of heat stress in poultry through thermal conditioning and nutritional manipulation. J Therm Biol.

[5] Alagawany M, Qattan S, Attia YA (2021). Use of chemical nano-selenium as an antibacterial and antifungal agent in quail diets and its effect on growth, carcasses, antioxidant, immunity and caecal microbes. Animals.

[6] Nawab A, Ibtisham F, Li G (2018). Heat stress in poultry production: Mitigation strategies to overcome the future challenges facing the global poultry industry. J Therm Biol.

[7] Li M, Wu J, Chen Z (2015). Effects of heat stress on the daily behavior of Wenchang chickens. Braz J Poult Sci.

[8] Fathi MM, Galal A, El-Safty S, Mahrous M (2013). Naked neck and frizzle genes for improving chickens raised under high ambient temperature: I. Growth performance and egg production. World’s Poult Sci J.

[9] Decuypere E, Huybrechts LM, Kuhn ER, Tixier-Boichard M, Merat P (1991). Physiological alterations associated with the chicken sex-linked dwarfing gene. Crit Rev Poult Biol.

[10] Yalcin S, Testik A, Ozkan S, Settar P, Celen F, Cahaner A (1997). Performance of naked neck and normal broilers in hot, warm, and temperate climates. Poult Sci.

[11] Lin H, Jiao HC, Buyse J, Decuypere E (2006). Strategies for preventing heat stress in poultry. World’s Poult Sci J.

[12] Duangjinda M, Tunim S, Duangdaen C, Boonkum W (2017). Hsp70 genotypes and heat tolerance of commercial and native chickens reared in hot and humid conditions. Braz J Poult Sci.

[13] Wang SH, Cheng CY, Tang PC (2015). Acute heat stress induces differential gene expressions in the testes of a broiler-type strain of Taiwan country chickens. PLoS ONE.

[14] Cedraz H, Gromboni JGG, Garcia AAP (2017). Heat stress induces expression of HSP genes in genetically divergent chickens. PLoS ONE.

[15] Yadav AK, Tomar SS, Jha AK, Singh J (2017). Importance of molecular markers in livestock improvement: A review. Int J Agric Innov Res.

[16] Hill WG (2012). Quantitative genetics in the genomics era. Curr Genomics.

[17] Falconer DS, Mackay TFC (1996). Introduction to quantitative genetics.

[18] Aksoy T, İlaslan Çürek D, Narinç D, Önenç A (2021). Effects of season, genotype, and rearing system on broiler chickens raised in different semi-intensive systems: performance, mortality, and slaughter results. Trop Anim Health Prod.

[19] Loengbudnark W, Chankitisakul V, Boonkum W (2023). The genetic impact of heat stress on the egg production of Thai native chickens (Pradu Hang dum). PLoS ONE.

[20] González Ariza A, Arando Arbulu A, Navas González FJ, Nogales Baena S, Delgado Bermejo JV, Camacho Vallejo ME (2021). The study of growth and performance in local chicken breeds and varieties: A review of methods and scientific transference. Animals.

[21] Padhi MK (2016). Importance of indigenous breeds of chicken for rural economy and their improvements for higher production performance. Scientifica.

[22] Adoligbe C, Fernandes A, Osei-Amponsah R (2020). Native chicken farming: A tool for wealth creation and food security in Benin. Int J Livest Prod.

[23] Halima H, Neser FWC, Van Marle-Koster E, De Kock A (2007). Village-based indigenous chicken production system in north-west Ethiopia. Trop Anim Health Prod.

[24] Baumgard LH, Rhoads RP (2013). Effects of heat stress on post-absorptive metabolism and energetics. Annu Rev Anim Biosci.

[25] Beede DK, Collier RJ (1986). Potential nutritional strategies for intensively managed cattle during thermal stress. J Anim Sci.

[26] Bell DD, Weaver WD (2002). Commercial chicken meat and egg production.

[27] Ewing SA, Lay DC, von Borell E (1999). Farm animal well-being-stress physiology, animal behavior, and environmental design.

[28] Novero RP, Beck MM, Gleaves EW, Johnson AL, Deshazer JA (1991). Plasma progesterone, luteinizing hormone concentrations, and granulosa cell responsiveness in heat-stressed hens. Poult Sci.

[29] Rozenboim I, Tako E, Gal-Garber O, Proudman JA, Uni Z (2007). The effect of heat stress on ovarian function of laying hens. Poult Sci.

[30] Elnagar SA, Scheideler SE, Beck MM (2010). Reproductive hormones, hepatic deiodinase messenger ribonucleic acid, and vasoactive intestinal polypeptide-immunoreactive cells in hypothalamus in the heat stress-induced or chemically induced hypothyroid laying hen. Poult Sci.

[31] Mashaly MM, Hendricks GL, Kalama MA, Gehad AE, Abbas AO, Patterson PH (2004). Effect of heat stress on production parameters and immune responses of commercial laying hens. Poult Sci.

[32] Deng W, Dong XF, Tong JM, Zhang Q (2012). The probiotic Bacillus licheniformis ameliorates heat stress-induced impairment of egg production, gut morphology, and intestinal mucosal immunity in laying hens. Poult Sci.

[33] Purswell JL, Dozier WA, Olanrewaju HA, Davis JD, Xin H, Gates RS Effect of temperature-humidity index on live performance in broiler chickens grown from 49 to 63 days of age.

[34] National Oceanic and Atmospheric Administration (1976). Livestock Hot Weather Stress.

[35] Mignon-Grasteau S (1999). Genetic parameters of growth curve parameters in male and female chickens. Br Poult Sci 1999; 40:44–51. Br Poult Sci.

[36] Bohmanova J, Misztal I, Tsuruta S, Norman HD, Lawlor TJ (2008). Short communication: Genotype by environment interaction due to heat stress. J Dairy Sci.

[37] Misztal I, Tsuruta S, Lourenco D, Aguilar I, Legarra A, Vitezica Z Manual for BLUPF90 Family of Programs.

[38] Ravagnolo O, Misztal I (2000). Genetic component of heat stress in dairy cattle, parameter estimation. J Dairy Sci.

[39] Chomchuen K, Tuntiyasawasdikul V, Chankitisakul V, Boonkum W (2022). Comparative study of phenotypes and genetics related to the growth performance of crossbred Thai indigenous (KKU1 vs. KKU2) chickens under hot and humid conditions. Vet Sci.

[40] Kim KG, Choi ES, Kwon JH, Sohn SH (2017). The effect of early chick weight on market-weight in Korean native chickens. Korean J Poult Sci.

[41] Tasonieroa G, Cullerea M, Baldan G, Zotte AD (2018). Productive performances and carcase quality of male and female Italian Padovana and Polverara slow-growing chicken breeds. Ital J Anim Sci.

[42] Tongsiri S, Jeyaruban GM, Hermesch S, van der Werf JHK, Li L, Chormai T (2019). Genetic parameters and inbreeding effects for production traits of Thai native chickens. Asian-Australas J Anim Sci.

[43] Promket D, Ruangwittayanusorn K (2021). The comparatives of growth and carcass performance of the Thai native chicken between economic selection (Chee KKU12) and natural selection (Chee N). Vet Integr Sci.

[44] Manjula P, Park HB, Seo D (2018). Estimation of heritability and genetic correlation of body weight gain and growth curve parameters in Korean native chicken. Asian-Australas J Anim Sci.

[45] Narinc D, Karaman E, Aksoy T, First MZ (2014). Genetic parameter estimates of growth curve and reproduction traits in Japanese quail. Poult Sci.

[46] Adeyinka IA, Oni OO, Nwagu BI, Adeyinka FD (2006). Genetic parameter estimates for body weights of naked neck broiler chickens. Int J Poult Sci.

[47] Saatchi M, Omed H, Dewi IA (2006). genetic parameters from univariate and bivariate analyses of egg and weight traits in Japanese quail. Poult Sci.

[48] Elston RC, Stewart J (1971). A general model for the genetic analysis of pedigree data. Hum Hered.

[49] Renaudeau D, Collin A, Yahav S, Basilio V, Gourdine JL, Collier RJ (2012). Adaptation to hot climate and strategies to alleviate heat stress in livestock production. Animal.

[50] Mutibvu T, Chimonyo M, Halimani TE (2017). Physiological responses of slow-growing chickens under diurnally cycling temperature in a hot environment. Braz Poult Sci J.

[51] Syafwan S, Kwakkel RP, Verstegen MWA (2011). Heat stress and feeding strategies in meat-type chickens. World’s Poult Sci J.

[52] Settar P, Yalcin S, Turkmut L, Ozkan S, Cahanar A (1999). Season by genotype interaction related to broiler growth rate and heat tolerance. Poult Sci.

[53] Al-Batshan HA (2002). Performance and heat tolerance of broilers as affected by genotype and high ambient temperature. Asian-Australas J Anim Sci.

[54] Aengwanich W (2007). Effects of high environmental temperature on blood indices of Thai indigenous chickens, Thai indigenous chickens crossbred and broilers. Int J Poult Sci.

